# Processing and Microstructure of As-Cast Ti-45Al-2W-xC Alloys

**DOI:** 10.3390/ma15145049

**Published:** 2022-07-20

**Authors:** Tomas Cegan, Kateryna Kamyshnykova, Juraj Lapin, Ivo Szurman, Jan Jurica, Vendula Klimantova

**Affiliations:** 1 Faculty of Materials Science and Technology, VSB—Technical University of Ostrava, 70800 Ostrava, Czech Republic; tomas.cegan@vsb.cz (T.C.); ivo.szurman@vsb.cz (I.S.); jan.jurica@vsb.cz (J.J.); vendula.klimantova@vsb.cz (V.K.); 2 Institute of Materials and Machine Mechanics, Slovak Academy of Sciences, Dubravska Cesta 9, 84513 Bratislava, Slovakia; kateryna.kamyshnykova@savba.sk

**Keywords:** intermetallics, TiAl, vacuum induction melting, carbides, solidification, microstructure

## Abstract

The metallurgical preparation and microstructure of as-cast Ti-45Al-2W-xC (in at.%) alloys were investigated. Five alloys with carbon content ranging from 0.38 to 1.96 at.% were prepared by vacuum induction melting (VIM) in graphite crucibles, followed by centrifugal casting into graphite moulds. A master 15W-85Al (at.%) alloy with a relatively low melting point and TiC powder were used to facilitate fast dissolution of W during VIM and to achieve the designed content of C in the as-cast alloys, respectively. The increase in the content of C affects the solidification path of the studied alloys. Differential thermal analysis (DTA) and microstructural observations show that the alloys with carbon content up to 0.75 at.% solidify with β primary phase and their dendritic as-cast microstructure consists of the α_2_(Ti_3_Al) + γ(TiAl) lamellar regions, retained B2 phase enriched by W and single γ phase formed in the interdendritic region. The increase in the content of C above 0.75 at.% leads to the formation of primary lathe-shaped Ti_2_AlC carbides, which act as effective heterogeneous nucleation sites of β dendrites during the solidification and grain refinement of the alloys with 1.15 and 1.96 at.% C. The increase in the content of C leads to an increase in Vickers hardness and elastic modulus in the alloys containing 1.96 at.% C.

## 1. Introduction

Among various high-temperature materials, lightweight TiAl-based alloys are of great interest due to their low density and a good combination of mechanical properties at elevated temperatures [1,2,3,4,5,6]. These materials can fulfil requirements for some structural applications in the automotive, energy, and aerospace industries. However, their low room temperature ductility, difficult processing, and insufficient creep resistance at temperatures higher than 750 °C limit their wider industrial use [7]. Refractory metals such as W with a low diffusivity in TiAl alloys can significantly increase mechanical properties and corrosion resistance at elevated temperatures [7]. Alloying with W reduces dislocation mobility and increases the thermal stability of lamellar microstructure, which leads to an increase in creep resistance [8,9,10,11,12,13]. The positive influence of carbon on the creep strength of TiAl-based alloys has been published by several authors [14,15,16,17]. The carbide precipitation is greatly influenced by carbon solubility limit, which varies with Al content and the content of other alloying elements [16,18,19]. The effect of carbon content on creep has been investigated mainly in Nb-containing TiAl-based alloys. It has been shown that the increase in creep strength of these alloys can be attributed to solid solution and precipitation strengthening mechanisms. The carbides forming in TiAl-based alloys belong to the cubic perovskite Ti_3_AlC (P-type) phase with needle-like morphology or to the hexagonal Ti_2_AlC (H-type) phase with plate-like morphology [20,21,22]. Above the carbon solubility limit in TiAl-based alloys, the formation of primary Ti_2_AlC carbides have been reported, for which chemistry, size, and shape depend on the alloying additions, carbon content, and processing parameters [23,24,25,26,27,28,29].

Vacuum induction melting (VIM) followed by precise gravity or centrifugal casting is identified to be one of the most cost-effective technologies for the production of complex-shaped components from TiAl-based alloys [30,31,32,33,34]. In recent years, additive manufacturing (AM) has been intensively explored as a new method for the fabrication of complex-shaped components from TiAl-based alloys [35,36,37]. The main factor limiting a wider application of additive manufacturing for the production of TiAl-based alloys and components is the availability and high price of TiAl powders with the required chemical composition [2]. Both VIM combined with precise casting and AM depend on the availability of TiAl ingots with the required chemical composition and quality. Up to now, several techniques such as vacuum arc remelting (VAR), plasma arc melting (PAM), electron beam melting (EBM), and VIM have been developed for the production of TiAl ingots [38,39]. Several different types of oxide ceramics have been tested for VIM of TiAl alloys, e.g., CaO, Al_2_O_3_, Y_2_O_3_, BaZrO_3_, and BaZrO_3_/Y_2_O_3_. It was found that the type of oxide ceramic material affects significantly the oxygen content and amount of non-metallic inclusions in the as-cast alloys [40,41,42,43,44,45,46]. VIM of TiAl-based alloys in graphite crucibles leads to an increase in the content of C, which can be beneficial for the improvement of high temperature creep resistance [17,47]. It has been shown that the content of C can be controlled by an appropriate selection and optimisation of processing parameters, such as heating rate, melt temperature, holding time at the melt temperature, arrangement of the charge in a graphite crucible, chemical composition of master alloys, and vacuum pressure [26,47,48]. Water-cooled copper crucibles have been applied for VIM or VAR to minimize contamination of TiAl ingots. However, heat losses due to significant heat dissipation due to cooling of the copper crucible significantly reduce energy efficiency of TiAl ingot production [49,50]. 

Kamyshnykova et al. [51] have reported that the mechanical properties of heat-treated TiAl–W based alloys can be improved by carbon additions ranging from 0.4 to 2 at.%. The Ti–45Al-2W-xC (at.%) alloys with fully lamellar microstructure and the addition of carbon close to its solubility limit have shown superior creep resistance compared to that of carbon-free TiAl–W and reference TiAl-based alloys at a temperature of 800 °C. A significant improvement in high-temperature creep resistance of Ti-45Al-2W-xC (at.%) alloys has been attributed to the solid solution strengthening effect of carbon, better thermal stability of α_2_ + γ lamellar microstructure, stabilisation of α_2_ phase with carbon, and precipitation strengthening by Ti_3_AlC and B2 particles forming during decomposition of the α_2_ phase [51]. Despite previous studies on the metallurgical processing of TiAl ingots by VIM in graphite crucibles [14,26,44,47,51,52] and the properties of W containing TiAl-based alloys [10,11,12,13], no information has been published on the effect of C on the microstructure of as-cast TiAl-W alloys.

This study aims to investigate the metallurgical processing route consisting of VIM in graphite crucibles followed by centrifugal casting into graphite moulds for the preparation of ingots with nominal composition Ti-45Al-2W-xC (at.%), where content of carbon ranges from x = 0.4 to 2 at.%. The effect of the content of C on the microstructure, segregation behaviour of alloying elements, phase transformations, hardness, and elastic modulus of five as-cast alloys is reported and discussed.

## 2. Materials and Methods

VIM was used for the alloy preparation. Scanning electron microscopy (SEM), thermo-evolution methods for determination of interstitial elements, X-ray difraction analysis (XRD), and energy dispersive spectroscopy (EDS) were used to investigate the chemical composition, phase identification, microstructure, and segregation. Differential thermal analysis (DTA) was used to determine the phase transition temperatures and hardness testing experiments were used for mechanical testing. Five independent measurements for each type of alloy were applied, with the exception of segregation profiles and DTA.

**Figure 1:**
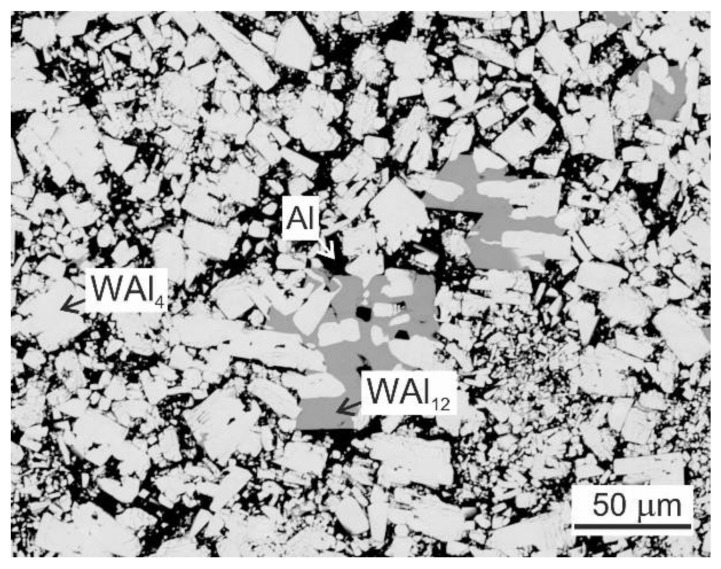
BSE micrograph of the microstructure of master 15W-85Al (at.%) alloy.

**Figure 2:**
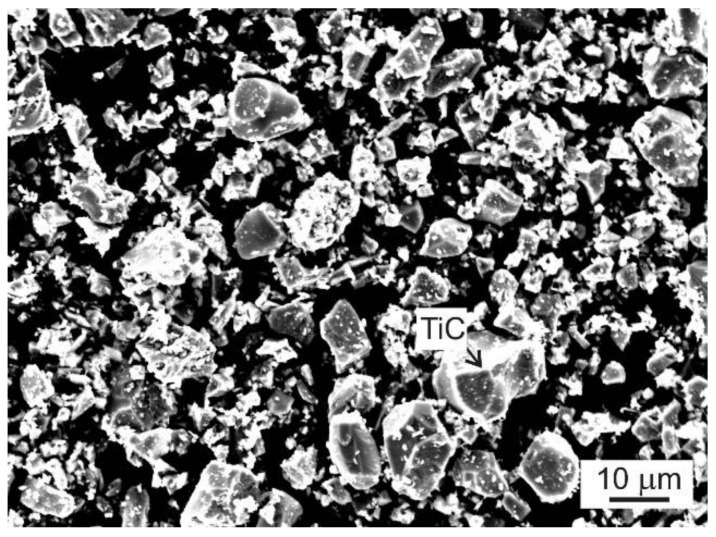
SEM micrograph of TiC powder.

**Figure 3:**
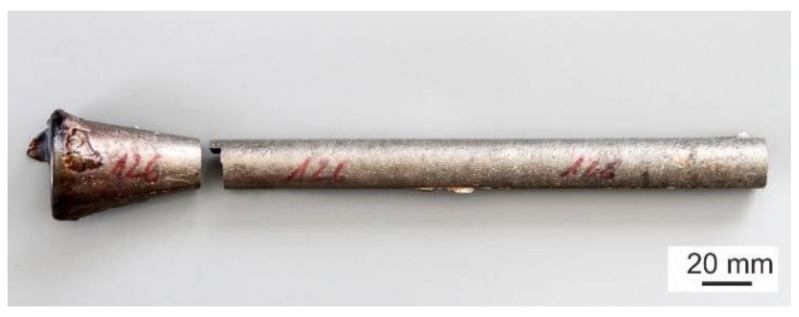
The typical example of the as-cast ingot from Ti-45Al-2W-xC alloy.

The studied TiAl-based alloys with designed nominal composition Ti-45Al-2W-(0.4–2)C (at.%) were prepared by VIM in isostatically pressed graphite crucibles and centrifugally cast into a graphite mould using a Supercast device (Linn High Therm GmbH, Eschenfelden, Germany). Pure Ti (grade 2) and Al (99.99%) and pieces of 15W-85Al (at.%) master alloy (see Figure 1) were used as the charge. The master alloy, composed of WAl_4_ and WAl_12_ phases distributed in the Al matrix, had a relatively low melting point of 1350 °C compared to that of pure W, which was used to facilitate the fast dissolution of W during VIM [53]. Isostatically pressed graphite crucibles ϕ65/135 mm (diameter/length) were applied to melt a charge with a weight of 410 g. An alumina-based crucible connected with a graphite mould was used to protect the graphite melting crucible. Before melting, the vacuum induction furnace was evacuated to a pressure of 20 Pa and rinsed with high purity Ar (99.9995%) three times. After increasing the vacuum pressure to 1.5 × 10^4^ Pa by a partial filling of the vacuum chamber with high purity argon, the charge was heated to a melt temperature of 1700 °C and held at this temperature for 30 s. The temperature of the melt was measured by a pyrometer. Different contents of carbon in the alloys were achieved by the addition of TiC powder into the charge, as shown in Figure 2. Figure 3 shows the typical example of an as-cast ingot with a diameter of 20 mm and length of 200 mm length in which the conical feeding head was removed by cutting using a diamond wheel.

Typical wet grinding using 60–2000 sandpapers (grains/cm^−2^) was used for metallographic sample preparation. Diamond suspensions (from 6 to 0.3 µm) were used for polishing the grounded surfaces of the samples. The microstructure evaluation was carried out with light microscopy (LM) and SEM using an Olympus GX 51 microscope (Olympus Corporation, Shinjuku, Japan) and a Quanta 450 FEG microscope (FEI Company, Fremont, CA, USA), respectively. Secondary electron (SE) and back-scattered electron (BSE) modes have been used for SEM. EDS was applied to determine the chemical composition. Calibration of EDS with the Ti_2_AlC standard was carried out for the measurements of the composition of coarse carbide particles. A Bruker D8 DISCOVER diffractometer (Bruker, Billerica, MA, USA) was used for XRD analyses. X-ray tube with a rotating Cu anode operating at 12 kW and parallel beam geometry with a parabolic Goebel mirror in the primary beam were used during XRD measurements. The O, N, and H contents in the alloys were specified by the thermo-evolution method with LECO ONH 836 instrument (LECO, Geleen, Netherland). The average content of C was detected by the thermo-evolution method on an ELTRA CS 2000 instrument (ELTRA, Haan, Germany). Differential thermal analysis (DTA), was performed with Setaram SETSYS 18TM experimental analyser (SETARAM Instrumentation, Caluire, France). Electroerosive cutting (EDM) was used to prepare DTA samples (3 × 3 × 3 mm) from the as-cast ingots. The surface of the samples was subsequently modified by wet grinding (600 grains/cm^−2^). Corundum crucibles were used for DTA experiments. Triple flushing with high purity Ar (99.99995) was applied. The heating and cooling cycles of DTA were carried out at a rate of 15 °C/min under Ar atmosphere. The DTA cooling curves were analysed to determine the phase transformation temperatures. Melting points of pure Pd and Ag (99.9995) were used for calibrations of DTA apparatus. The weights of the individual samples and the cooling rates were taken into account for the correction of the phase transformation temperatures.

Hardness was measured by Zwick/Roell ZHU 2.5 equipment (ZwickRoell GmbH, Ulm, Deutschland). Instrumented Martens hardness measurements (98.1 N applied load, 1 s time, 15 N/s loading rate) were carried out. An applied load of 98.1 N and a time of load application of 10 s were used for Vickers hardness measurements.

## 3. Results and Discussion

### 3.1. Chemical Composition and Microstructure of the As-Cast Ingots

The studied alloys with nominal compositions Ti-45Al-2W-xC, where x is 0.4, 0.5, 0.8, 1.2, and 2.0 (in at.%) and designated as C4, C5, C8, C12, and C20, respectively, are summarized in Table 1. As can be seen in the table, the measured chemical compositions of the as-cast ingots, including the content of C, are close to the designed nominal ones. The measured high content of nitrogen up to 500 wt.ppm and oxygen up to 1200 wt.ppm in the ingots results from a lower purity of Ti containing up to 300 wt.ppm of N and up to 2500 wt.ppm of O. The present results indicate that VIM in graphite crucibles, followed by casting into graphite moulds, can be applied for the processing of TiAl-based alloys containing strong carbide forming elements such as Ti and W with the controlled carbon content and reproducible chemical composition. The applicability of VIM in graphite crucibles for the cost-effective processing of carbon-containing TiAl-based alloys with a controlled content of C and reproducible chemical compositions has been reported by several authors [14,25,47,54]. 

**Figure 4:**
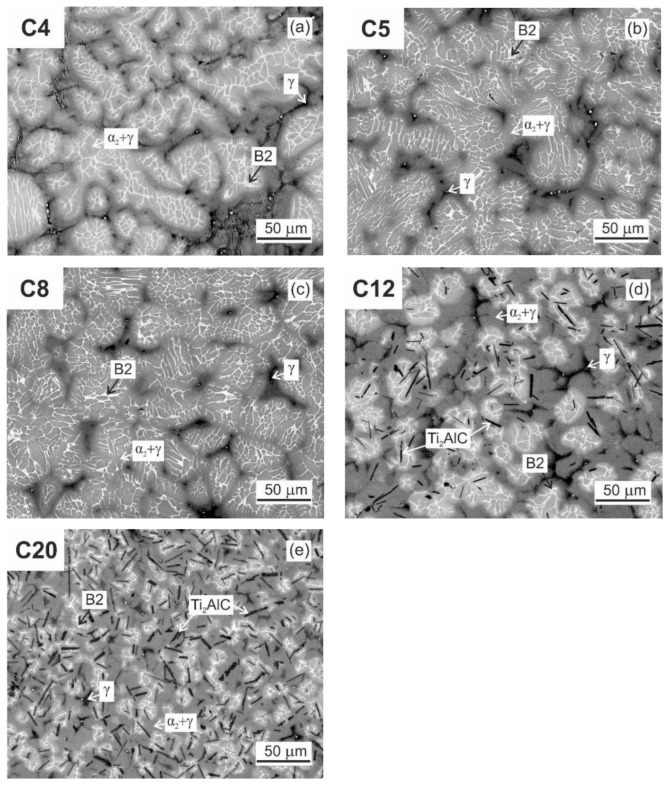
BSE micrographs showing the typical microstructue of the as-cast alloys: (**a**) C4; (**b**) C5; (**c**) C8; (**d**) C12; (**e**) C20.

**Figure 5:**
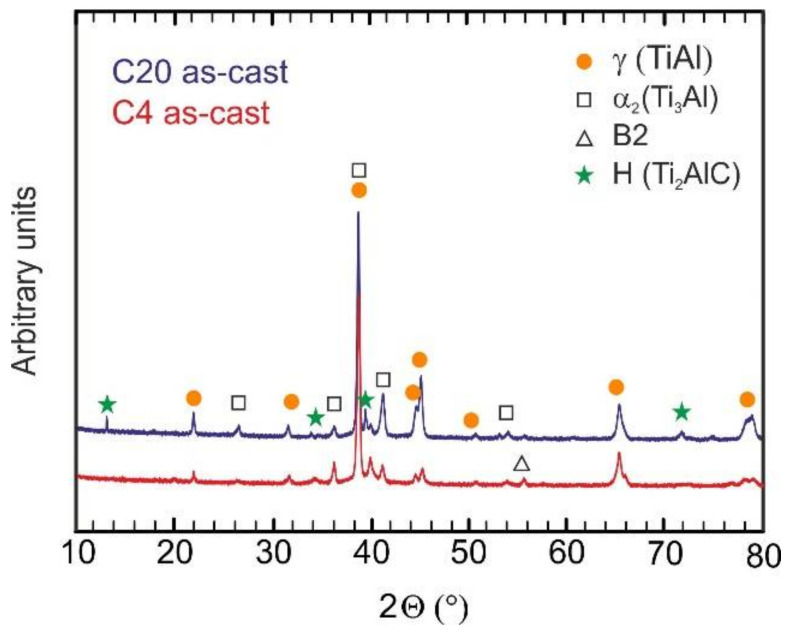
The typical X-ray diffraction patterns of C4 and C20 alloys.

**Figure 6:**
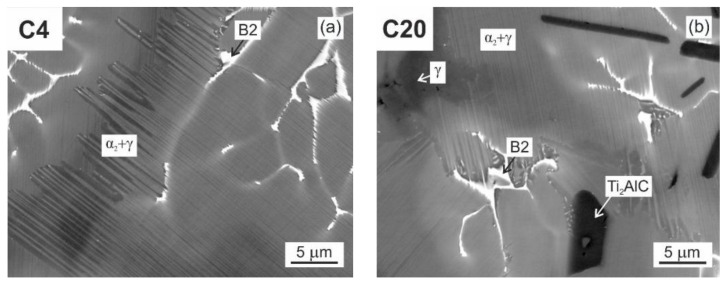
BSE micrographs showing the microstructure of as-cast alloys: (**a**) C4 alloy; (**b**) C20 alloy.

**Figure 7:**
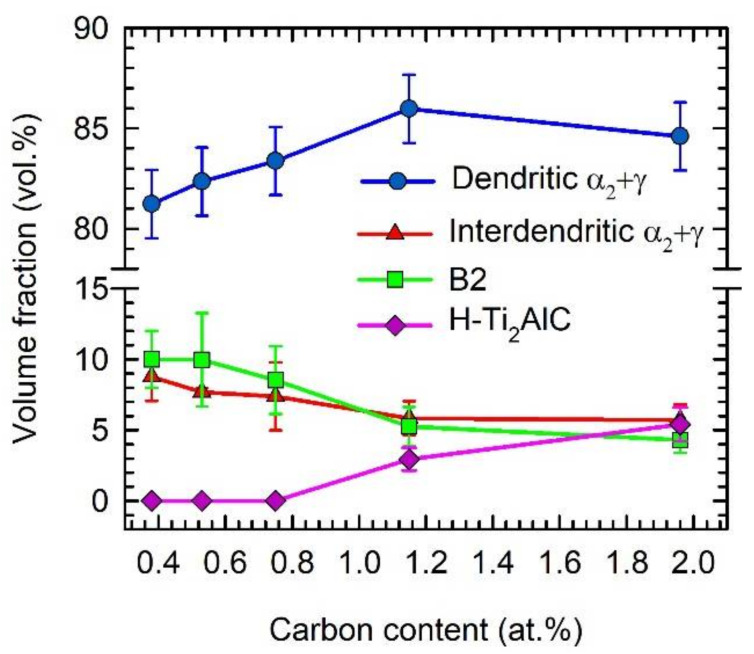
Dependence of the volume fraction of individual phases and microstructural regions on carbon content.

Figure 4 shows the typical microstructure of the as-cast C4, C5, C8, C12, and C20 alloys. The microstructure of the as-cast alloys is dendritic, as shown in Figure 4a–e. Within the dendrites, a bright network of the B2 phase results from the solid-state phase transformation of the β phase to the α and reordering of β to B2 at lower temperatures [55,56,57]. The α phase transforms into a fine lamellar α_2_ + γ microstructure and a low amount of a single γ phase can be preserved in the interdendritic region. This type of microstructure is typical for as-cast TiAl-based alloys, containing additions of Nb, Ta, Mo, and W [58,59]. However, the microstructure of the as-cast alloys shows significant changes with increasing carbon content. Figure 5 shows the typical XRD diffraction patterns of the C4 and C20 alloys, indicating the presence of four phases: γ, α_2_, B2, and H-Ti_2_AlC (in C20 alloy). Besides the C20 alloy, the formation of plate-like Ti_2_AlC particles is also identified in the C12 alloy, as shown in Figure 4d. Figure 6 shows the microstructure in the interdendritic region of the C4 and C20 alloys. In both alloys, the interdendritic region is composed mainly of the γ phase and a small amount of lamellar α_2_ phase, as shown in Figure 6a,b. Table 2 summarises the measured chemical composition of the individual phases and microstructural regions in the as-cast alloys. The individual phases and regions in the C4, C5, C8, C12, and C20 alloys do not show significant differences in the content of Ti, Al, and W. Figure 7 shows the dependence of the volume fraction of individual phases and microstructural regions on carbon content. The volume fraction of the B2 phase decreases from 10 vol.% to 4 vol.%, with increasing carbon content from 0.38 to 1.96 at.%. The same tendency is also observed for the volume fraction of the interdendritic α_2_ + γ region, which decreases from 8.8 vol.% to 6 vol.%. The volume fraction of dendritic lamellar α_2_ + γ regions increases with increasing carbon content from 81 vol.% to 85 vol.% with increasing carbon content from 0.38 to 1.96 at.%. It should be noted that the carbon content affects interlamellar α_2_-α_2_ spacing, which decreases with increasing carbon content up to a solubility limit of carbon in TiAl-based alloys [54]. The volume fraction of plate-like primary Ti_2_AlC particles increases from 2.9 vol.% to 5.4 vol.% with increasing carbon content from 1.15 to 1.96 at.%. As shown by Lapin et al. [23], the volume fraction of primary Ti_2_AlC particles depends on the carbon content and increases linearly with increasing carbon in Ti-44.5Al-8Nb-0.8Mo-0.1B-xC (at.%) alloys.

### 3.2. Segregation Behaviour of Alloying Elements

**Figure 8:**
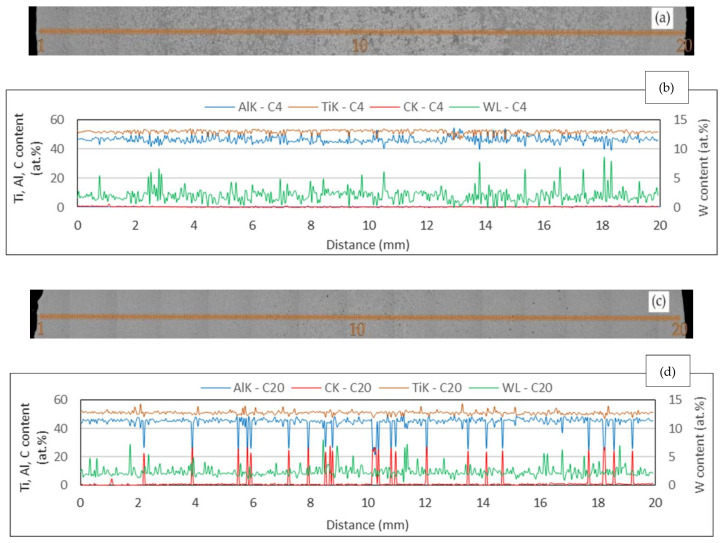
EDS concentration profiles of the alloying elements: (**a**) position of measurement points during the line analysis at a transversal section of the C4 ingot; (**b**) concentration profile of elements in the C4 ingot; (**c**) position of measurement points during the line analysis at a transversal section of the C20 ingot; (**d**) concentration profile of elements in the C20 ingot.

The segregation behaviour of the alloying elements and the chemical homogeneity of the as-cast ingots was assessed by the EDS line analysis with a step of 40 µm (500 points). Figure 8 shows the measured variations in the alloying elements with the illustration of the positions of the analyzed points at the transversal sections of the as-cast C4 and C20 ingots. Significant dendritic segregation can be observed in the sample C4 at first glance. The contents of Ti and Al in the C4 ingot vary from 50 to 53 at.% and from 44 to 47 at.%, respectively, as seen in Figure 8a,b. The most significant segregation is associated with a decrease in the content of Ti to 46 at.%. For Al, the most pronounced segregation is associated with the increase in the content of Al to 54 at.% in several analysed points, which is associated with the presence of an interdendritic single γ phase in the microstructure. The formation of the interdendritic γ phase in the as-cast alloys can be explained by the microsegregation behaviour of the alloying elements. Aluminium characterized by the partition coefficient of *k*_Al_ = 0.9 segregates preferentially into interdendritic liquid during solidification [60,61]. Locally, the last liquid enriched in Al solidifies through the formation of a single γ phase, as seen in Figure 4a–d [4,54]. It is important to note that O (*k*_O_ = 1.29) behaves similarly to Ti (*k*_Ti_ = 1.14) or W and segregates into the dendrites [60,61]. The EDS line analysis of the C20 ingot shows more pronounced segregation, with the opposite tendency associated with an increase in C content up to 26 at.% and a decrease in Al content to 25 at.%, as shown in Figure 8c,d. These segregation peaks result from the presence of the Ti_2_AlC particles in the microstructure. The content of W in both analyzed samples is relatively stable (1–3 at.%), and more pronounced segregation is caused by the occurrence of the B2 phase enriched in W and interdendritic γ phase enriched locally in Al. A significantly lower occurrence of larger interdendritic regions containing a single γ phase and chemically more homogenous structure of the intermetallic matrix indicates a change in solidification behaviour of the C20 alloy, which will be discussed later.

### 3.3. Formation of Primary Carbide Particles

**Figure 9:**
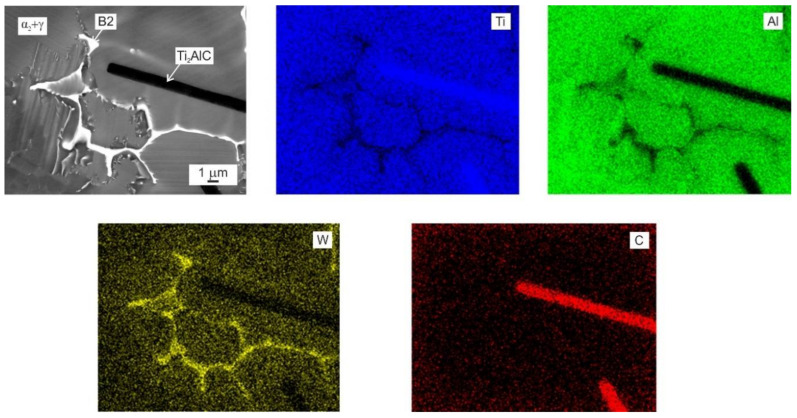
BSE micrograph and corresponding EDS map analysis of the as-cast C20 alloy. The analysed elements such as Ti, Al, W, and C are marked in the EDS map figures.

Figure 9 shows the BSE micrograph and corresponding EDS mapping of elemental distribution in the reinforcing carbide particles and the surrounding matrix of the C20 alloy. An increase in Ti and C and a decrease in Al and W contents can be seen in the primary Ti_2_AlC particles. Although W is a strong carbide-forming element, it segregates preferentially into the remaining β/B2 phase, stabilizing this high-temperature phase forming during the solidification in the microstructure of the as-cast alloys, as seen in Figure 9. The very low solubility of W in the primary Ti_2_AlC (layered hexagonal MAX phase) particles has also been reported by Lapin and Kamyshnykova [25] for Ti-45Al-5Nb-2W-2C (at.%) alloy. It should be noted that the MAX phase forming elements such as Nb (Nb_2_AlC type of MAX phase) and Ta (Ta_2_AlC type of MAX phase) show high solubility, but the solubility of Mo and W is very limited in Ti_2_AlC phase [23,26,57].

Table 2 shows that Ti_2_AlC particles in the C12 and C20 alloys contain 46.7–47.2 at.% Ti, 25.4–25.6 at.% Al, 0.8 at.% W and 26.6–26.9 at.% C. The measured W content in the carbides is likely affected by the surrounding matrix. The Ti and Al contents have a ratio close to 2:1, which is typical for the Ti_2_AlC type of carbide and is in agreement with the publications of several authors [16,23,26,27,28,29]. Since only the alloys with 1.15 (2.9 vol.%) and 1.96 at.% C (5.4 vol.%) contain homogeneously distributed primary lathe-shaped Ti_2_AlC particles, the solubility limit of C in the studied Ti-45Al-2W-xC alloys is between 0.75 and 1.15 at.%, which is a higher interval than that ranging from 0.5 to 0.75 at.% reported by Gabrisch et al. [21] for Ti-45Al-5Nb-xC (at.%) alloys.

The average width and length of the primary carbides are measured to be (1.8 ± 1.1) µm and (10.6 ± 6.6) µm for the C12 alloy and (2.9 ± 1.9 µm) and (14.8 ± 10.3) µm for C20 alloy, respectively. The formation of primary Ti_2_AlC particles in the C12 and C20 alloys can be explained by assuming the Ti-Al-C phase diagram reported by Witusiewicz et al. [62]. The melt temperature of 1700 °C used in the present study corresponds to L(liquid) + TiC_1−x_ phase region. During cooling, the TiC_1−x_ particles transform to plate-like Ti_2_AlC according to a transformation pathway L + TiC_1−x_ → L + Ti_2_AlC. The solidification continues by the formation of a secondary phase which nucleates preferentially on the plate-like Ti_2_AlC particles present in the melt. The plate-like Ti_2_AlC particles serve as heterogeneous nucleation sites during solidification, which leads to the microstructure refinement as described for similar carbon-containing TiAl-based alloys in the as-cast state by several authors [16,23,25,26,29]. The presence of the primary carbides in the C12 and C20 alloys leads to the refinement of the dendritic structure, as shown in Figure 4d,e. The refinement of the dendritic structure due to the formation of the primary Ti_2_AlC carbides leads to a significant reduction in the grain size. For the C4, C5, and C8 alloys, the typical columnar grains with an average length of (200 ± 70) µm and diameter of (90 ± 35) µm are observed in the microstructure. For the C12 alloy containing 2.9 vol.% of Ti_2_AlC, the average length and diameter of columnar grains are measured to be (70 ± 40) µm and (40 ± 10) µm, respectively. For the C20 alloy containing 5.4 vol.% of Ti_2_AlC, the average length and diameter of columnar grains are measured to be (45 ± 20) µm and (30 ± 15) µm, respectively, which means a reduction of grain length by up to 80% and width by up to 70% compared to samples without primary carbides.

### 3.4. DTA Analysis of As-Cast Alloys

**Figure 10:**
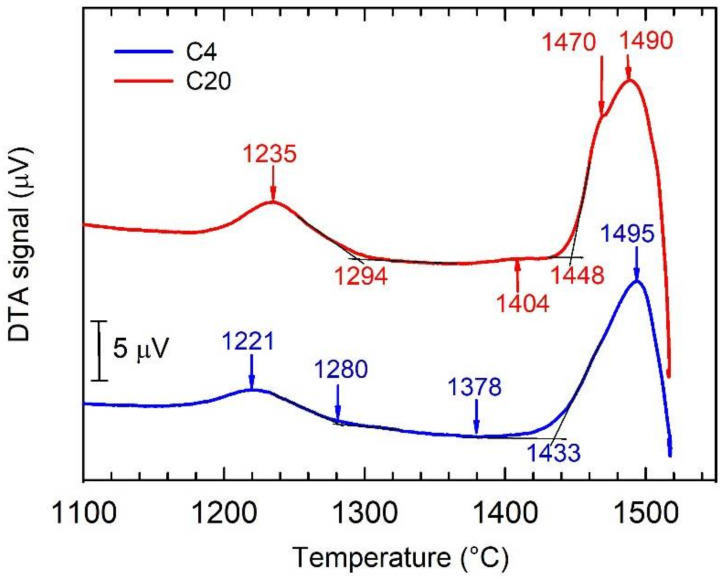
DTA cooling curves for the C4 and C20 alloys.

Figure 10 shows the DTA cooling curves of the C4 and C20 alloys. The phase transformation sequences and corresponding temperatures determined from the DTA cooling curves are summarized in Table 3. The solidification of the C4 alloy starts with the formation of the primary β phase. The β phase transforms into the α phase following the transformation sequence β → β + α in a temperature range between 1495 and 1433 °C. Figure 4a indicates that this transformation is incomplete and the network of untransformed β phase enriched by W is preserved in the as-cast microstructure. A similar type of microstructure has been reported by Ding et al. [5] for Nb-containing TiAl-based alloys solidifying through the β phase and subsequent solid-state phase transformation to α phase without a peritectic reaction/transformation. The single α phase is stable in a temperature range from 1433 to 1378 °C. Assuming the pseudo-binary Ti-Al phase diagram calculated for 2 at.% W up to a temperature of 1300 °C [63] and the fact that 0.38 at.% C does not affect significantly the phase equilibria, the formation of a single α phase region is expected. In the temperature range from 1378 to 1280 °C, the α phase transforms into a lamellar microstructure according to the transformation sequence α → α + γ. The decrease in temperature from 1280 to 1221 °C leads to an instability of the two-phase microstructure according to the transformation sequence α + γ → α + γ + β. According to the phase diagram [63], at temperatures below 1221 °C, one should expect the formation of thermodynamically stable microstructure following the transformation sequence α + γ + β → γ + B2, which has not been observed in the as-cast alloy.

In the C20 alloy, the solidification starts with the formation of primary H-Ti_2_AlC particles, which is followed by the solidification of the β phase according to the transformation sequence L + H → β + L + H [23]. The DTA cooling curve for the C20 alloy indicates that the β + L + H phase-field region is stable in a temperature range from 1490 to 1470 °C. At a temperature of 1470 °C, a peritectic reaction/transformation following a transformation sequence β + L + H → β + α + H leads to a full solidification of the studied C20 alloy. The presence of peritectic reaction/transformation can also be seen in Figure 4e. The peritectic α phase is formed around the β dendrites, which leads to a significant reduction in the amount of the untransformed β enriched by W and the interdendritic regions enriched by Al, as seen in Figure 4e and Figure 8b. In the temperature range from 1470 to 1448 °C, the β transforms to the α phase according to the transformation sequence β + α + H → α + H. The α + H phase is stable at temperatures between 1448 and 1404 °C. The decrease in temperature from 1404 to 1294 °C leads to an instability of the α phase according to the transformation sequence α + H → α + γ + H. In the temperature range between 1294 and 1235 °C, β phase is formed according to the transformation sequence α + γ + H → α + γ + β + H. It is expected that at temperatures below 1235 °C, a thermodynamically stable microstructure should be formed according to a transformation sequence α + γ + β + H → γ + B2 + H, which has not been achieved in the as-cast C20 alloy.

### 3.5. Hardness and Elastic Modulus

**Figure 11:**
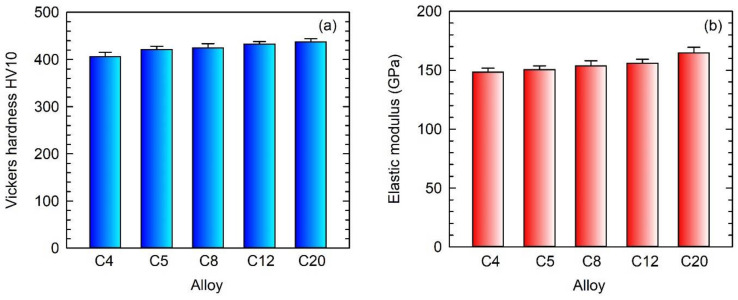
Vickers hardness and elastic modulus of the as-cast C4, C5, C8, C12, and C20 alloys: (**a**) Vickers hardness HV10; (**b**) Indentation elastic modulus. The studied alloys are marked in the figure.

Figure 11 shows average values of Vickers hardness HV10 and indentation elastic modulus measurements in the as-cast C4, C5, C8, C12, and C20 alloys. It should be noted that the average values are calculated from 20 independent measurements carried out for each alloy. The increase in C content in the ingots leads to an increase in Vickers hardness HV10, as seen in Figure 11a. This increase in HV10 can be attributed to solid solution strengthening of both α_2_ and γ phases by carbon [19,53,64]. The solubility of carbon in the γ phase and α_2_ phase was determined to be 0.025–0.25 at.% [18,64] and about 1 at.% [19,65], respectively. Thus, the α_2_ phase can dissolve a much higher amount of carbon. However, the solubility of carbon in coexisting phases strongly depends on the chemical composition of the alloy. The increase in hardness of carbon-containing TiAl-based alloys can be attributed mainly to the solid solution strengthening effect of interstitial carbon atoms and, to a lesser extent, to the precipitation hardening with Ti_3_AlC and Ti_2_AlC carbide particles [21]. The C4 alloy shows the lowest average value of HV10, which can be attributed to the highest volume fraction of the soft interdendritic γ phase [57] in the microstructure (see Figure 6) and the lowest solid solution hardening of the coexisting phases by interstitial atoms of C. The formation of hard primary Ti_2_AlC particles [66] contributes to higher hardness values of the C12 and C20 alloys compared to those of C4, C5, and C8 ones. All variations in the indentation elastic modulus of the C4, C5, C8, and C12 alloys fall into the experimental error of the measurements, as seen in Figure 11b. Statistically significantly higher elastic modulus is measured in the C20 alloy. The increase in the elastic modulus to 165 GPa in the C20 alloy can be attributed to the presence of 5.4 vol.% of primary Ti_2_AlC particles with an elastic modulus of 234–250 GPa [25,57], which is higher than those of 176 GPa, 144 GPa, and 190 GPa reported for TiAl, Ti_3_Al, and B2 phases, respectively [66,67,68].

## 4. Conclusions

The metallurgical preparation of TiAl-based alloys with nominal composition Ti-45Al-2W-xC, where x = 0.4, 0.5, 0.8, 1.2, and 2 (at.%), and the effect of the content of C on their microstructure, segregation behaviour of alloying elements, phase transformations, hardness, and elastic modulus were studied. The following conclusions are reached:

1. The use of master 15W-85Al (at.%) alloy facilitated a fast dissolution of W in the melt and led to the metallurgical preparation of Ti-45Al-2W-(0.4–2) C alloys with designed and reproducible chemical composition, using VIM in graphite crucibles and centrifugal casting into a graphite mould.
2. The microstructure of the as-cast alloys was dendritic, with high segregation of W to the dendrites. Within the dendrites, a network of B2 phase surrounded by α phase was formed, which transformed into fine lamellar α_2_ and γ phases during cooling. The interdendritic regions mainly contained the γ phase and a small amount of α_2_ phase.
3. The alloys with additions of 1.15 and 1.96 at.% C contained coarse lathe-shaped Ti_2_AlC particles. The solubility limit of C in the studied alloys was determined to be between 0.75 and 1.15 at.%. The Ti_2_AlC carbides dissolve only a low amount of W, which segregates preferentially into β dendrites during solidification.
4. The Ti_2_AlC particles acted as heterogeneous nucleation sites during solidification, significantly refining the dendritic microstructure and reducing the grain size. The volume fraction of interdendritic α_2_ + γ regions and B2 phase decreased with increasing carbon content in the as-cast alloys.
5. The DTA and microstructural analysis showed that the alloys with carbon content up to 0.75 at.% solidify through β primary phase. The alloys with the carbon content above 1.15 at.% solidify through Ti_2_AlC primary phase. The increase in carbon content up to 1.15 at.% changed significantly on the solidification path, from L → L + β to L + H → L + β + H.
6. The Vickers hardness increases with the increasing content of C in the as-cast alloys. The elastic modulus of the C20 alloy with 5.4 vol.% of primary Ti_2_AlC particles is higher than that measured in the C4, C5, C8, and C12 alloys.

## Figures and Tables

**Table 1:** Chemical composition of prepared alloys.

Alloy	Al (at.%)	Ti (at.%)	W (at.%)	O (wt.ppm)	N (wt.ppm)	C (at.%)
C4	44.7 ± 0.4	52.9 ± 0.3	2.0 ± 0.2	1120 ± 50	500 ± 20	0.38 ± 0.03
C5	45.2 ± 0.3	52.2 ± 0.3	2.1 ± 0.1	1130 ± 15	460 ± 25	0.53 ± 0.02
C8	44.9 ± 0.3	52.3 ± 0.3	2.0 ± 0.1	910 ± 15	370 ± 10	0.75 ± 0.02
C12	44.7 ± 0.4	52.1 ± 0.4	2.0 ± 0.1	1190 ± 75	480 ± 25	1.15 ± 0.02
C20	44.5 ± 0.5	51.5 ± 0.5	2.0 ± 0.1	1010 ± 20	400 ± 25	1.96 ± 0.03

**Table 2:** Chemical composition of individual regions in the as-cast C4, C5, C8, C12, and C20 alloys.

Alloy	Phases	Al	Ti	W	C
C4	B2	38.4 ± 0.4	55.2 ± 1.0	6.40 ± 0.08	-
	α_2_ + γ	43.9 ± 0.3	53.0 ± 0.3	3.10 ± 0.03	-
	γ	50.3 ± 0.8	49.4 ± 0.6	0.30 ± 0.02	-
C5	B2	39.1 ± 0.4	54.4 ± 1.3	6.60 ± 0.07	-
	α_2_ + γ	43.9 ± 0.2	53.4 ± 0.2	2.70 ± 0.03	-
	γ	51.2 ± 0.6	48.2 ± 0.8	0.60 ± 0.02	-
C8	B2	39.3 ± 0.9	53.5 ± 0.8	7.20 ± 0.09	-
	α_2_ + γ	44.2 ± 0.3	53.3 ± 0.3	2.50 ± 0.04	-
	γ	51.7 ± 0.6	47.7 ± 0.6	0.60 ± 0.03	-
C12	B2	39.4 ± 0.7	53.3 ± 0.9	7.30± 0.07	-
	α_2_ + γ	43.7 ± 0.4	54.1 ± 0.5	2.20 ± 0.04	-
	γ	50.9 ± 0.4	48.7 ± 0.4	0.40 ± 0.05	-
	H	25.4± 0.6	47.2 ± 0.2	0.80 ± 0.02	26.6 ± 0.4
C20	B2	38.9 ± 0.8	53.8 ± 0.9	7.40 ± 0.08	
	α_2_ + γ	44.5 ± 0.3	52.8 ± 0.3	2.70 ± 0.03	
	γ	50.4 ± 0.4	49.0 ± 0.5	0.60 ± 0.03	
	Ti_2_AlC	25.6 ± 0.5	46.7 ± 0.2	0.80 ± 0.03	26.9 ± 0.4

**Table 3:** Overview of the phase transformation sequences and corresponding temperatures determined from DTA cooling curves.

Sample	Phase Transformation	Temperature (°C)
C4	β → β + α	1495
	β + α → α	1433
	α → α + γ	1378
	α + γ → α + γ + β	1280
	α + γ + β → γ + B2	1221
C20	β + L + H	1490
	β + L + H → β + α + H	1470
	β + α + H → α + H	1448
	α + H → α + γ + H	1404
	α + γ + H → α + γ + β + H	1294
	α + γ + β + H → γ + B2 + H	1235

## Data Availability

The data are stored by the authors of the paper, not available publically.
